# Mindfulness and yoga therapy for acute pain in sickle cell disease

**DOI:** 10.1002/jha2.819

**Published:** 2023-11-15

**Authors:** Pallavi Dev, Natalie Bavli, Brittney Sims, Jenny Foster, Anna Moscowitz, Una E. Makris, Siayareh Rambally

**Affiliations:** ^1^ Department of Internal Medicine UT Southwestern Medical Center Dallas USA

## Abstract

There is a paucity of data regarding the use of non‐pharmacologic therapies for pain in sickle cell disease. The purpose of this pilot study was to assess the acceptability and feasibility of video‐guided mindfulness meditation, breathing exercises, and yoga, in addition to standard of care, during admission for painful vaso‐occlusive crisis. Feasibility was demonstrated by the enrollment rate of > 90% and high level of participant engagement in the intervention. Acceptability was demonstrated by positive feedback obtained in post‐intervention surveys and the majority of subjects who expressed interest in participating in future mindfulness and yoga therapy sessions.

## Graphical abstract

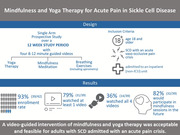

## 1 INTRODUCTION

Sickle cell disease (SCD) is a group of hereditary hemoglobinopathies that results in both acute and chronic pain, poor quality of life, organ damage, and early mortality. Painful vaso‐occlusive crisis (VOC) is the leading cause of hospitalization and morbidity among patients with SCD [1]. It is estimated that 18%–59% of patients with SCD experience five or more VOCs per year. VOCs are most often managed with opioids; however, these medications have significant side effects and often provide inadequate relief. Patients with SCD may have central sensitization to pain, which contributes to opioid‐induced hyperalgesia, allodynia, and neuropathic pain [2]. By adulthood, the majority of patients with SCD experience pain on more than half of their days, with nearly one‐third reporting pain almost every day [3]. Chronic pain results in changes in synaptic networks that increase sensitivity to pain and link the sensation to negative emotions. Chronic pain has been closely associated with anxiety, depression, fatigue, poor functional status, and social isolation, which contribute to worse health‐related quality of life outcomes. A more comprehensive, biopsychosocial approach to pain management is warranted in adults with SCD to address the multiple mechanisms from which their pain emerges [2, 4].

Yoga has proven therapeutic benefits in chronic pain syndromes by altering the perception of and attitude toward pain [5]. Additionally, yoga may alleviate anxiety and depression, which are highly prevalent in the sickle cell population and closely intertwined with the subjective experience of physical pain [6]. Mindfulness‐based meditation, which enhances awareness of the present moment, is an integral aspect of yoga therapy [7]. Acceptance of the present moment may decrease suffering in those struggling with physical pain, thus facilitating the healing process [8, 9].

Few studies have investigated the benefits of yoga and mindfulness‐based interventions in the SCD population [10, 11]. The American Society of Hematology 2020 guidelines offer a conditional recommendation based on “very low certainty in the evidence” for yoga therapy as an adjunctive management strategy for acute pain in SCD. The guidelines do not offer a recommendation for or against “combined meditation/movement programs” for chronic pain related to SCD. Yoga and mindfulness programs for pain in SCD have been identified as areas requiring further research [12].

The purpose of this study was to assess the acceptability and feasibility of video‐guided mindfulness meditation and yoga therapy, in addition to standard‐of‐care treatments, for the management of VOCs in hospitalized patients with SCD.

## 2 METHODS

### 2.1 Population and recruitment

A single‐arm prospective study of yoga therapy, mindfulness meditation, and breathing exercises (including the use of incentive spirometry) was conducted in adult patients with SCD who were experiencing a VOC requiring hospitalization. This study was approved by an institutional IRB and all patients completed an informed consent prior to enrollment.

Our recruitment period was a pre‐specified 12‐week interval during which we sought to include as many eligible SCD patients admitted with a VOC. The inclusion criteria were adult patients, age 18 and older, with SCD (any genotype), admitted to a single large academic hospital with a VOC during the study period. This included pregnant patients and those with organ complications of SCD. Patients were excluded if they were unable to consent or follow simple instructions. Patients in the intensive care unit were excluded due to the severity of their condition.

### 2.2 Intervention and delivery

Videos used in this study were filmed by the UT Southwestern media team and featured a certified yoga therapist as well as study team members. Each of the four videos was approximately 8–12 min in duration and focused on guided meditation, breathing exercises (including the use of an incentive spirometer), and gentle movement that could be performed from a supine position in a hospital bed. Participants were encouraged to modify movements based on their energy level, ability, and comfort. After the participants were shown how to access the videos on the screen in their hospital room, they were free to select and watch the videos of their choosing at their own pace. The participants were able to pause or stop the videos at any time.

### 2.3 Outcomes

The feasibility of this intervention was assessed by the proportion of sickle cell patients approached over the 12‐week study period who agreed to participate, as well as participant engagement in the intervention once enrolled. Participant engagement was determined by objective data captured from the video‐based platform, including the number of videos viewed and the percentage completed. A video was considered completed if more than 85% of the video was viewed. A post‐intervention multiple‐choice survey was administered to assess the acceptability of the intervention and willingness to participate in future sessions. The study team also conducted in‐person structured interviews to obtain open‐ended feedback (see Table 2). Demographic data was collected from the patient's medical record (see Table 1).

**TABLE 1:** Demographics and participation.

Demographics	
Total # of participants	39
Male	15 (38.5%)
Female	24 (61.5%)
Age in years, median (range)	31 (27–43)
Length of hospital stay in days, median (range)	8 (6–11)
Genotype	
Hgb S S	24 (61.5%)
Hgb S C	8 (20.5%)
Hgb S β0 thal	5 (12.8%)
Hgb S β+ thal	1 (2.6%)
Hgb S O Arab	1 (2.6%)

Intervention Engagement	
Number of videos watched per participant	
Median/IQR	2 (1–4)
Mean/SD	2.1 (1.6)
Number of participants who watched	
Zero videos	8 (20.5%)
One video	8 (20.5%)
Two videos	8 (20.5%)
Three videos	1 (2.6%)
Four videos	14 (35.9%)
Number of views per video	
*Nourishing Your Joints with Gentle Movements* (guided mindful movements ending in meditation to relax tension)	15 (38.5%)
*Leading Life with Your Heart* (use of incentive spirometry to guide deep breathing and calming tapping technique)	22 (56.4%)
*Breathing Toward Relaxation and Healing Blue Light Meditation* (box breathing and blue light meditation)	23 (59.0%)
*Breath Awareness* (diaphragmatic breathing and body scan meditation)	23 (59.0%)

### 2.4 Statistical analysis

This was a pilot project to assess the feasibility and acceptability of this novel intervention over a prespecified 12‐week period, therefore, the sample size was not calculated. Descriptive statistics were used to calculate baseline demographic data. Continuous and categorical variables were expressed as medians with interquartile ranges and counts with percentages, respectively.

## 3 RESULTS

Over a 12‐week period, 42 patients were approached for the study and 39 agreed to participate. The median age of our participants was 31. Most were female (62%), and 100% were African American. The median length of hospital stay was 8 days (interquartile range 6–11).

Seventy‐nine percent of participants (31 of 39) watched at least one video. Among those who participated in the intervention, 45% (14 of 31) completed all four videos. On average, participants viewed two videos. Most patients (72%) found the yoga and mindfulness practices helpful, and twenty‐seven participants (69%) enjoyed the sessions. Patients who did not watch any videos (*n* = 8) cited feeling tired, difficulty focusing, distractions, lack of motivation, forgetting, and pain as barriers. Those who found the sessions helpful often used terms associated with relaxation and calmness to describe their experience.

Thirty‐two (82%) of the participants expressed a desire to participate in future mindfulness and yoga therapy sessions, with interest in both in‐person and virtual platforms (see Table 2). Suggestions to improve the sessions included minimizing interruptions, expanding options for mobility in the exercises, adding elements that would further promote feelings of calmness (such as music), and enabling access to the sessions after discharge home.

**TABLE 2:** Survey questions and responses (*n* = 39).

Written multiple‐choice survey questions	
Did you find the mindfulness practices helpful?	
Yes	28 (71.8%)
No	2 (5.1%)
Neutral or didn't participate	9 (23.1%)
Did you enjoy participating in the sessions?	
Yes	27 (69.2%)
No	2 (5.1%)
Neutral or didn't participate	10 (25.6%)
Would you participate in mindfulness sessions in the future?	
Yes	32 (82.1%)
No	3 (7.7%)
Neutral or didn't participate	4 (10.3%)

Structured interview questions	
Did you participate in the mindfulness sessions during this hospitalization?	
Yes	32 (82.1%)
No	7 (17.9%)
If not, what prevented you from doing so?	Selected quotes (from five responses): “People kept coming in and out so it was hard to focus.” “I saw one but didn't participate. When patients are in pain it's hard to go the extra mile to do the exercises.” “Was up at night and too tired during the day.”
Did the mindfulness and yoga therapy sessions help you during this hospitalization?	
Yes	29 (74.4%)
No	3 (7.7%)
No answer	7 (17.9%)
If yes, in what ways did they help you?	Selected quotes (from 27 responses): “The breathing sessions were very helpful. I will take what I learned from the breathing for my delivery. It also helped my pain.” “It helped calm my mind from overthinking. Helped me get my mind off the pain.” “Tapping helped with calming me down the most.” “It helped me relax and focus on other things besides pain.” “Slept really good.” “It helped with pain intensity, zeroing on where the pain was and breathing through it, [and] put me in a relaxed state.”
How did you feel after completing a mindfulness session?	Selected positive quotes (from 29 responses): “Felt a calm, warm sensation.” “Like a breath of fresh air. Relaxed.” “Felt really good and at peace.” “Relief, peaceful, but pain is still present.” “Relaxed, open, and clear mind.” “Refreshed.” Selected neutral or negative quotes (from 3 responses): “No particular feelings.” “I didn't feel anything after completing.”
Do you have any recommendations on how we can improve the sessions?	Selected quotes for suggestions on ways to improve (from 11 responses): “Could be more options for yoga outside of bed.” “Add calm music.” “It can be hard to do movements when hurting.” “Would like to have access to it at home.” No suggestions (27 responses)
Would you prefer in‐person or virtual sessions? Individual or group sessions?	
In‐person	6 (20.7%)
Virtual	11 (37.9%)
Both/either in‐person, virtual	12 (30.8%)
Individual	7 (25%)
Group	7 (25%)
Both/either individual, group	14 (50%)

## 4 DISCUSSION

This study demonstrated that video‐guided mindfulness and yoga therapy are both acceptable and feasible for patients with SCD admitted with a VOC, as demonstrated by the high enrollment rate (93%), participation rate (79%), percentage of patients who found the practices helpful (72%), and percent interested in future participation (82%). The intervention is low risk to patients. It is also low‐cost to the health care system; once the videos are developed, they can be distributed to multiple hospitalized patients over an unlimited time frame.

In 2021, a pilot study of virtual group yoga therapy sessions in the outpatient setting conducted by our research team demonstrated high rates of mental health conditions among participants: 100% with moderate or severe depression via the PHQ‐9 scale, 78% with moderate or severe anxiety via the GAD‐7 scale, and high rates of pain catastrophizing (93^rd^ percentile via the pain catastrophizing scale). Unfortunately, participation was low due to technical difficulties and scheduling conflicts with the one‐hour weekly sessions. However, this study demonstrated patient interest in continuing mindfulness and yoga therapy in the outpatient setting. The best outcomes are likely to occur with more consistent practice, so overcoming barriers to outpatient participation should be a future area of focus.

A strength of this study is that it uses the inpatient setting as an opportunity to present this pilot intervention to patients and gather feedback. Another strength is the broad inclusion criteria that enhance generalizability among patients with different sickle cell genotypes and clinical presentations. The downside to the inpatient setting is that the acuity of illness may potentially distract from engagement in the intervention. Several limitations are worth noting. This pilot study was conducted at one academic hospital setting in Texas, so results may not apply to other geographic locations or clinical settings. Another limitation is that no outcome measures related to pain, mental health, or quality of life were obtained. Now that feasibility and acceptability have been demonstrated in this pilot study, assessing these outcome measures can be priorities of future research.

## 5 CONCLUSION

Acute and chronic pain experienced by patients with SCD is complex and multi‐factorial. It has become increasingly clear that a holistic approach, including non‐pharmacologic therapies that address psychosocial wellbeing, is necessary to improve the quality of life of our patients. In this pilot study, we demonstrated the feasibility and acceptability of mindfulness‐based interventions and yoga therapy for sickle cell patients in the acute inpatient setting. Future directions include expanding access to the outpatient setting and assessing outcome measures for quality of life, pain, and mental health.

## AUTHOR CONTRIBUTIONS

Pallavi Dev, Natalie Bavli, Una E. Makris, and Siayareh Rambally contributed to the design and implementation of the research study, data analysis, and written manuscript. Brittney Sims and Anna Moscowitz contributed to the implementation of the research study. Jenny Foster contributed to the written manuscript.

## CONFLICT OF INTEREST STATEMENT

Dr. Una E. Makris reports funding by VA Health Services Research and Development (HX003350) and NIA (P30 AG022845) for research unrelated to this work/manuscript with no other conflict of interest.

## FUNDING INFORMATION

This study received no funding external to the UT Southwestern Department of Hematology and Oncology.

## ETHICS STATEMENT

All subjects gave their informed consent for inclusion before they participated in the study. The study protocol was approved by the UT Southwestern Institutional Review Board (IRB) (Study # STU‐2021‐0729).

## CLINICAL TRIAL REGISTRATION

This study has been registered with ClinicalTrials.gov (ID # NCT05572294).

## Data Availability

The data that support the findings of this study are available from the corresponding author upon reasonable request.
